# Models of high-grade serous carcinoma of tubo-ovarian origin

**DOI:** 10.3389/or.2026.1849834

**Published:** 2026-07-15

**Authors:** Achuth Padmanabhan, Shailendra Dwivedi, Melissa A. Merritt, Priyanka Verma, Ronald J. Buckanovich, Jessica D. Lang, Hannah Dimmick, Kenneth P. Nephew, Ronny Drapkin, Lindsay W. Brubaker, Benjamin G. Bitler

**Affiliations:** 1 Department of Biological Sciences, University of Maryland Baltimore County, Baltimore, MD, United States; 2 University of Maryland Greenebaum Comprehensive Cancer Center, Baltimore, MD, United States; 3 Peggy and Charles Stephenson Cancer Center, The University of Oklahoma Health Sciences, Oklahoma City, OK, United States; 4 Department of Obstetrics and Gynecology, the University of Oklahoma Health Sciences, Oklahoma City, OK, United States; 5 The Daffodil Centre, The University of Sydney, and Cancer Council NSW, Sydney, NSW, Australia; 6 Faculty of Medicine and Health, The University of Sydney, Sydney, NSW, Australia; 7 Division of Oncology, Department of Medicine, Washington University in St. Louis, St. Louis, MO, United States; 8 University of Pittsburgh School of Medicine, Department of Medicine, and Department of Obstetrics, Gynecology and Reproductive Sciences, Pittsburgh, PA, United States; 9 Women’s Cancer, Magee-Womens Research Institute, Pittsburgh, PA, United States; 10 Department of Pathology and Laboratory Medicine, University of Wisconsin-Madison, Madison, WI, United States; 11 Center for Precision Medicine, University of Wisconsin-Madison, Madison, WI, United States; 12 Department of Obstetrics and Gynecology, University of Colorado Anschutz Medical Campus, Aurora, CO, United States; 13 Melvin and Bren Simon Comprehensive Cancer Center, Indiana University School of Medicine, Indianapolis, IN, United States; 14 Medical Sciences Program and Department of Anatomy, Cell Biology and Physiology, Indiana University School of Medicine, Bloomington, IN, United States; 15 Penn Ovarian Cancer Research Center, Perelman School of Medicine, University of Pennsylvania, Philadelphia, PA, United States; 16 Basser Center for BRCA, Abramson Cancer Center, University of Pennsylvania, Philadelphia, PA, United States; 17 Division of Gynecologic Oncology, Department of Obstetrics and Gynecology, University of Colorado Anschutz Medical Campus, Aurora, CO, United States; 18 Division of Reproductive Sciences, Department of Obstetrics and Gynecology, University of Colorado Anschutz Medical Campus, Aurora, CO, United States

**Keywords:** cell lines, high-grade serous, models, organoids, ovarian cancer, organoid, organ-on-a-chip

## Abstract

Biological models aim to recapitulate the molecular and histologic characteristics, as well as the carcinogenesis of the disease of interest, to enable high-fidelity experimentation and impactful translational development. In high-grade serous carcinoma of ovarian, tubal, or peritoneal origin, there is a need to regularly re-evaluate the experimental models being employed to address research questions. This review aims to provide a resource for better understanding the applications, strengths, and limitations of the available models. This work discusses the origin and carcinogenesis of high-grade serous carcinoma and reviews normal cell-of-origin models, as understanding the cellular origin is critical to experimental efforts focused on pathogenesis, disease progression, and prevention. High-grade serous carcinoma cell lines are discussed, including essential and complementary molecular features, cell line management, and limitations. Next, the review examines advances in organoids, with a specific focus on patient-derived organoids from ascites and single-cell suspensions, organ-on-a-chip platforms, and tissue slice technologies. Finally, given the heterogeneity of epithelial ovarian cancer in general, and high-grade serous carcinoma in particular, and the evolving understanding of the importance of the tumor microenvironment, *in vivo* models capture organism-level complexity. In this review, we focused on high-grade serous carcinoma. In this context, we review patient-derived xenograft (PDX), syngeneic, and genetically engineered mouse models. Each has its own advantages and disadvantages, and guidance is provided on the optimal use of these *in vivo* approaches for the study of high-grade serous carcinoma.

## Graphical Abstract

**Figure:**
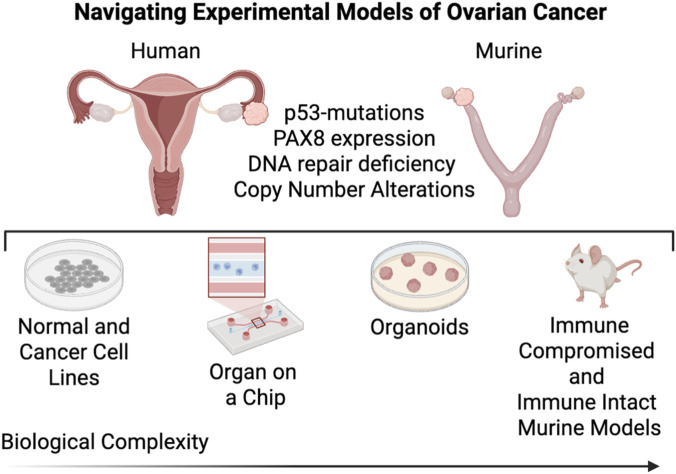

## Introduction

The most faithful attribute of any biological model, such as cell lines, organoids, or *in vivo* models, is its molecular fidelity to the organ of origin and the tumor subtype it aims to represent. This molecular fidelity is particularly important for addressing the heterogeneity in genomic, transcriptomic, cellular capabilities, and histopathological characteristics. As the gynecologic oncology research community continues to investigate and define ovarian cancer biology, specifically in the context of high-grade serous carcinoma (HGSC) of tubo-ovarian origin, there is a critical need to regularly re-evaluate the models being employed to answer essential ovarian cancer-related research questions.

The understanding of HGSC origin has evolved substantially. Although HGSC has historically been classified as ovarian, fallopian tube, or primary peritoneal based on anatomic presentation, accumulating human pathologic and murine modeling studies support the distal fallopian tube epithelium, particularly secretory epithelial cells and STIC-associated lesions, as the predominant precursor site for many HGSCs (1–4). Studies of the tubal-peritoneal junction and spatial analyses of precursor lesions further support a fallopian tube-associated model of early serous carcinogenesis (5, 6). However, experimental CRISPR-modified organoid models indicate that both the fallopian tube epithelium and the ovarian surface epithelium can give rise to tumors with high-grade serous features under specific genetic conditions, supporting a dual-origin model in selected contexts (7). Therefore, we have removed the peritoneum as a proposed epithelial cell of origin and clarified that “primary peritoneal HGSC” is best understood as an anatomic/clinical designation rather than as evidence of mesothelial origin.

In normal development, the fallopian tubes arise from the Müllerian ducts and are characterized by their expression of the transcription factor Paired Box 8 (PAX8) (8–10). Consequently, most high-grade serous carcinoma tumors overexpress PAX8 (11, 12). Fallopian tube epithelial cells undergo a transformation characterized by a “p53 signature,” typically observed as a largely intact columnar epithelial architecture with either loss of p53 expression or an accumulation of stabilized, mutated p53 (1, 13). Some p53 signature lesions further progress to serous tubal intraepithelial carcinoma, characterized by loss of epithelial architecture and increased proliferation. A recent review and research publication highlight the pathologic and transcriptomic changes during HGSC progression (14, 15). Transformed cells from serous tubal intraepithelial carcinoma lesions are proposed to exfoliate onto the ovary and/or into the peritoneal cavity, survive in an anchorage-independent environment, and seed distant sites such as the adipocyte-rich omentum, leading to a diagnosis of advanced-stage high-grade serous carcinoma (16, 17).

When selecting an experimental model, molecular similarity to patient tumors, rather than tissue of origin alone, is critical for defining a representative high-grade serous carcinoma model. Many models are available that match patients’ tumor mutational and expression profiles, making them biologically relevant models of high-grade serous carcinoma; the optimal model strives to recapitulate the mutational pattern and carcinogenesis of HGSC. Several reviews also detail the molecular, genetic, and growth features of HGSC and non-HGSC models (18–22). The objective of this review is to provide a reference for investigators during the selection of biological models to further understand ovarian cancer biology, specifically high-grade serous carcinoma. We explore cell lines, organoids, and *in vivo* models to identify the cells of origin of high-grade serous carcinoma and discuss the limitations of these models and opportunities for further innovation.

## Methodology

This review represents an expert-curated synthesis of the literature focused on experimental models of high-grade serous carcinoma (HGSC) of tubo-ovarian origin. Relevant studies were identified through searches of PubMed/MEDLINE, and Google Scholar using combinations of keywords: “high-grade serous carcinoma,” “HGSC,” “fallopian tube epithelium,” “serous tubal intraepithelial carcinoma”, “ovarian cancer cell lines,” “patient-derived organoids,” “tumor slice culture,” “organ-on-a-chip,” “patient-derived xenograft,” “humanized mouse models,” “syngeneic models,” “genetically engineered mouse models”, “cancer-associated fibroblasts,” “tumor-associated macrophages,” “T cell exhaustion,” “single-cell RNA sequencing,” “spatial transcriptomics,” “hypoxia,” “metabolism,” “ascites spheroids”, “PARP inhibitor resistance,” “platinum resistance,” “immune evasion”. ChatGPT Plus was used to assist with organizing the literature and editing the language; all scientific content and references were independently reviewed and verified by the authors. Priority was given to primary research articles between 1974 and 2026. Studies were selected for relevance to HGSC, with an emphasis on molecular fidelity (e.g., TP53 alteration, chromosomal instability, homologous recombination status), histopathologic features, and applicability to biological questions, including tumor initiation, progression, metastasis, immune interactions, and therapeutic response. Inclusion criteria encompassed studies that described or validated HGSC-relevant models or provided mechanistic or translational insight, while exclusion criteria included studies lacking a clear histotype definition, those focused on non-epithelial ovarian cancers without HGSC relevance, or reports with limited methodological rigor.

## CELL lines

### Normal cells of origin models: ovarian surface and fallopian tube epithelium

Recent innovations in cell lines derived from putative normal cells of origin present exciting opportunities to advance our understanding of critical events in the initiation of high-grade serous carcinoma. The need to conduct comparative studies of both normal human ovarian surface and fallopian tube epithelial cells represents a key challenge in the study of the normal cells of origin for high-grade serous carcinoma. Local IRB approval is required to obtain normal human ovaries or normal fallopian tubes during surgery for non-malignant gynecological disorders, such as risk-reducing salpingo-oophorectomy. This is challenging as the distal fallopian tube, which is believed to be the primary source of high-grade serous carcinoma, is typically analyzed *in toto* by pathology for precursor lesions. As such, fresh tissue is often unavailable, or only the proximal fallopian tube is available for research.

From normal human ovaries, ovarian surface scrapings can be collected to establish cultures of ovarian surface epithelium, or slices can be cultured as explants. Once the epithelial cells grow out of the specimen, they can be collected. It is also necessary to perform immunocytochemistry for cytokeratin and vimentin on the passaged cells and confirm that the cells are positive for both markers. Furthermore, at least three independent primary human ovarian surface epithelial cell cultures from different subjects are required to ensure rigor and reproducibility. In addition, immortalized human ovarian surface epithelial cell lines have been established by ectopic expression of hTERT and p53 shRNA (23) or by expression of human papillomavirus oncogenes E6/E7 (24). However, there is no difference in culture conditions for immortalized human ovarian surface epithelium and primary human ovarian surface epithelial cells.

Several methods have been described to create immortalized human secretory fallopian tube epithelial cell lines for use in large-scale experiments, including the use of retroviruses to stably express hTERT, p53 shRNA, and CDK4^R24C^ (25). This approach has been adopted by other laboratories, while some studies have developed protocols and culture conditions for primary, non-transformed fallopian tube epithelial cells (26, 27).

Other studies have described two-dimensional (2D) *in vitro* culture models for primary human ovarian surface and fallopian tube epithelial cells, but were unable to culture normal ovarian surface epithelial cells (28) or normal fallopian tube epithelial cells (29) for more than 12 population doublings using traditional culture media. In 2013, a chemically defined cell culture medium containing growth factors (EGF, insulin), cholera toxin, and hydrocortisone was described (WIT-fo, later renamed FOMI medium) to support the long-term propagation of normal ovarian surface or fallopian tube epithelial cells (30). Additionally, a series of paired normal ovarian surface and fallopian tube epithelial cells from the same cancer-free donors was used to derive a set of cell lines representing immortalized cells (expressing ectopic hTERT) and transformed derivatives (after sequential introduction of SV40T/t antigen and H-Ras) (30).

Understanding the normal initiation of fallopian tube and ovarian surface epithelium precursors is crucial to achieving the goals of preventing HGSC and detecting HGSC at an early stage. Similarly, there is a need for modeling the precursor lesions of HGSC, including the p53 signature and serous tubal intraepithelial carcinoma (STIC) lesions. Although ‘omics data, including spatial transcriptomic information, have provided significant insights into the molecular mediators of these lesions, replicable human models of STIC lesions and an appropriate *in vitro* model for STIC present a major challenge in studying its pathogenesis. However, several recent efforts have used 3D culture techniques to develop organoid models that originate from the normal human fallopian tube epithelium (hFTE). For example, fallopian tube organoids (FTO) and CRISPR-Cas9 gene editing were used to generate FTO with *TP53* loss-of-function mutations (31). Cellular and molecular characterization revealed that *TP53* ^*−/−*^ FTOs mimicked key STIC features (31). Human FTO with TP53 plus RAD51D mutations replicated an early-stage HGSC phenotype *in vitro* and affected sensitivity to platinum and PARPi (32). A 3D multicompartment “assembloid” model was developed that resembles the fallopian tube in molecular, functional, architectural, and physiological terms (33). Finally, using induced pluripotent stem cells (iPSCs) from young ovarian cancer patients with germline pathogenic BRCA1 mutations (BRCA1mut), Yucer et al. generated an FTO model that recapitulated BRCA1mut ovarian carcinogenesis in both vitro and *in vivo* (34). Thus, human-derived FTE organoid models that mimic STIC lesions together with other *ex-vivo* transplant 3D models (see recent review by Clark et al. and references included therein (35) are exciting developments that show great promise for improving the current understanding of the pathophysiology of precursor lesions to HGSC and potential for identifying specific biomarkers of early FTE cell transformation for early detection/preventative strategies, serving as biologically relevant platforms for evaluating drug responses *in vitro* and *in vivo* and personalized therapy.

### High-grade serous carcinoma (HGSC) cell lines

Several high-quality research publications and reviews outline the current state of ovarian cancer cell lines (18–21, 36) and serve as resources for understanding the criteria for cell line designation, including the assays and technologies used to classify cell line histotype and assess *in vivo* growth of different cell lines in mice (37–39).

Historically, when considering and selecting cell lines, the most defining feature has been the molecular profile. HGSC tumors are often characterized by mutation, loss, or repression of tumor suppressor genes. For instance, in HGSC tumors, nearly all cases exhibit mutations in the *TP53* gene. Even in HGSC tumors that express wild-type p53 (∼2%), p53 activity was often deemed dysfunctional (40). HGSC tumors also have a loss of *NF1* (10%–20%), *RB1* (∼15%), or *PTEN* (∼5%) in a mutually exclusive manner (41). The loss of tumor suppressor genes is typically identified through DNA sequencing, but this approach does not capture epigenetically silenced genes. For example, PTEN, while deleted in 5% of tumors, has RNA expression below the median in an additional 10% of tumors (41, 42). In addition, approximately 50% of tumors are considered DNA repair-deficient due to mutations or epigenetic repression of DNA double-strand break repair factors, including BRCA1/2, PALB2, and RAD51C/D (43–46). While HGSC has a high propensity for tumor suppressor inactivation, oncogene activation is also observed, including the amplification of MYC (∼30%) (47), FOXM1 (∼7%) (41), and CCNE1 (∼20%) (41, 48), although the sequence and significance of additional tumor suppressor inactivation or oncogenic activation remain to be fully elucidated.

The aberrant molecular features observed in HGSC often lead to marked chromosomal instability, which is reported as copy number changes or loss of heterozygosity, although HGSC tumors rarely have elevated tumor-associated point mutation burden compared to other types of cancer, such as melanoma (HGSC: 88.2 mutations/tumor vs. melanoma: 740.6 mutations/tumor) (41, 49). To aid investigators in selecting appropriate HGSC cell lines, Table 1 summarizes essential and complementary HGSC molecular features, along with a technique for measuring each feature. As noted in Table 1, several distinguishing molecular features include PAX8 expression, CCNE1 amplification, homologous recombination DNA repair status, and responses to platinum-based chemotherapy and PARP inhibitors. Notably, these features, including genetic dependencies, are available for most HGSC cell lines via DepMap (50, 51). The molecular profiles of HGSC models have continued to be refined over the last 20 years. For instance, multiple primary research articles and reviews have revisited the histological designation of a panel of ovarian cancer cell lines (18–21, 36), which serve as an excellent resource for selecting histotype-appropriate cell lines. The histotype designations for 88 commonly used ovarian cancer cell lines are listed in Supplementary Table S1.

**TABLE 1:** Molecular features of high-grade serous carcinoma cells. IHC = immunohistochemistry.

Characteristic	Feature	Technique
Essential	TP53 Mutation/TP53 dysfunction	Sequencing/IHC
	PAX8 expression	IHC
Additional	Chromosomal instability (1q, 3q, 8q, 12p, 19q Amp) (5q, 16q, 17q Loss)	Exome sequencing Low-pass whole genome sequencing
	Limited point mutations (<100)	Genome sequencing
	Survival in anchorage independent context	*In vitro* experimentation
	Double strand break repair deficiency BRCA1/2-mutation or downregulation PALB2 mutation RAD51C/D mutation ATM/ATR mutation	RAD51-foci formation Targeted sequencing RNA-seq inference
	NF1 mutation loss or mutation	Targeted sequencing
	PTEN mutation or downregulation	Targeted sequencing/RNA-seq

As therapy resistance is commonly observed in ovarian cancer, several groups have reported derivatives of cell lines that match therapy-sensitive and resistant versions. For instance, Hoare et al described the generation of platinum-resistant OVCAR4, OVSAHO, and COV318 cell lines, which demonstrated a non-genetic mechanism of resistance (52). These matched lines are powerful models for further defining the biological processes underlying therapy resistance. In addition, various functional assays, including sensitivity to platinum and poly(ADP-ribose) polymerase (PARP) inhibitors, were performed on a panel of HGSC (37) cell lines to further guide their applications and improve understanding of their molecular drivers.

With respect to HGSC cell lines, studies have shown that cell lines like SK-OV-3 (p53 mutated, HRAS and ARID1A-mutated, ERBB2 amplification) (53, 54), OVCAR5 (p53 mutated, KRAS mutated) (55), and A2780 (p53 wildtype, PIK3CA and ARID1A-mutated) (21, 56) do not entirely align with key genetic alterations as discussed above. Additionally, other cell lines, such as OVCAR8, TYK-nu, and HEYA8, are considered ovarian in nature but may represent a histotype other than HGSC (57). Additionally, lab-derived BRCA2 reversion mutations have been observed in PEO1 cells (58), and are still commercially sold in the United States, necessitating careful consideration of BRCA2 status of this model. Notably, cell lines and their designated histotypes are often based on only a single or a few studies. In some cases, purely bioinformatic analyses are being used to determine or reassign histotypes; however, when isolated from functional evaluation, these approaches are limited. For instance, ovarian cancer cell lines with homologous recombination deficiency (HRD) are exquisitely sensitive to PARP inhibitors. Recently, the widely used OVCAR3 cell line (p53-mutant, CCNE1-amplified) was observed to be sensitive to PARP inhibitors despite lacking overt HRD features (i.e., *BRCA1/2* mutations or other mutations in homologous recombination repair) (59). This highlights the importance of complementing molecular features with functional testing. Thus, in assigning histotype to ovarian cancer cell lines, efforts should be made to develop uniform and standardized strategies for assigning their respective histotypes, such as the empirical numerical scoring method proposed by Domcke et al. (21). Overall, selecting models with robust molecular and functional validation is crucial for ensuring consistent and reproducible results.

Additionally, it is important to note that HGSC transcriptional subtypes (proliferative, mesenchymal, immunoreactive, and differentiated) are reproducibly identified in bulk datasets but do not strictly reflect tumor cell–intrinsic states (41). Rather, emerging evidence indicates that subtype classification is largely driven by tumor cellular composition, with stromal and immune infiltration shaping mesenchymal and immunoreactive signatures, respectively (60). Accordingly, these subtypes are best interpreted as tumor ecosystem states. This distinction has important implications for model selection; epithelial-only systems are most appropriate for studying tumor-intrinsic programs, whereas modeling mesenchymal and immunoreactive states requires systems that incorporate fibroblasts, immune cells, or an *in vivo* microenvironmental context. These models and features are discussed in more detail below.

### CRISPR/Cas9 editing of ovarian cancer cell lines

The intended use of a cell line is another important factor for optimal selection. For example, while *BRCA1/2* mutations are less common in HGSC (*BRCA1/2* germline mutations 13%–22% and somatic mutations 6%–12%) (41, 61), the development of molecularly faithful *BRCA*-mutant HGSC cell lines is critical for developing more precise treatment strategies and risk profiles. *BRCA1/2*-mutated or HRD HGSC cell lines have been widely used in functional genomic screens (e.g., CRISPR/Cas9 knockout screens) to examine genetic dependencies and identify new therapeutic vulnerabilities (17, 62). However, CRISPR/Cas9 gene editing and large genomic screens rely on stable Cas protein expression. Given that the Cas protein introduces DNA double-strand breaks, stable expression of this nuclease is not feasible in many cell lines with *BRCA1*/*2* mutations due to increased propensity to induce apoptosis in response to DNA damage in HRD cells. Alternative approaches to overcome this biological limitation are 1) doxycycline-inducible systems to offer temporal control of Cas expression or 2) the use of nucleofection to transiently introduce the Cas9 protein with CRISPR single-guide RNAs. The challenge with this approach is that nucleofection conditions vary by cell line, and optimal nucleofector kits and programs must be identified individually for each cell line. Table 2 describes *BRCA*-mutant ovarian cancer cells, including their ability to be stably engineered to express Cas protein and the optimized nucleofection conditions. The noted conditions are optimal starting points when employing CRISPR/Cas-mediated gene editing (63). In some mechanistic studies, CRISPR-mediated gene knock-in can be useful to modify the genetic sequence in a specific manner. HRD status of HGSC cell lines is critically important to consider for gene knock-in applications, as homologous recombination is required to incorporate the donor template into the sequence.

**TABLE 2:** Nucleofection conditions for *BRCA1* and *BRCA2* mutated cell lines. del = deleted.

Cell line name	BRCA-mutation	Can be engineered to stably express Cas9?	Nucleofection protocol (Kit; Pulse code)
Primary ovarian cell	Any mutation	Context Dependent	P1; EA-104
BRCA1-mutated ovarian cancer cell lines			
UWB1.289^103^	BRCA1: p.D825EfsTer21; expresses hypomorphic BRCA1 del 11q (788-4096 nucleotide)^104^	Yes	SF; FE-132
UWB1.289+BRCA1^103^	Full length BRCA1 complemented back in UWB1.289	Yes	SF; FE-132
JHOS2	Predicted to have p.M1V mutation. Expression of BRCA1 not detected	No	SF; FE-132
JHOS4	BRCA1: C. 5278-1G>A (splice site mutation in intron 20)	Yes	SF; FE-132
COV362	BRCA1: p.F872VfsTer31; loss of complete protein expression	No	SF; FE-132
BRCA2-mutated ovarian cancer cell lines			
OVSAHO	BRCA2 homologous deletion; complete loss of BRCA2 expression	Yes	SF; FE-132
Kuramochi	BRCA2: c.6952C>T, p.R2318X; complete loss of BRCA2 expression	No	SF; DS-130
PEO1^51^	BRCA2: 5193C>G (Y1655X); complete loss of BRCA2 expression	No	SF; FE-132
PEO4^51^	Reversion BRCA2 mutation in Pe01 5193C>T (Y1655Y); restored BRCA2 expression	Yes	SF; FE-132

The CRISPR modality should be matched to both the model system and the biological question. Stable Cas9 pooled knockout screens are best suited for HR-proficient, molecularly faithful HGSC-like cell lines when the goal is to identify non-essential genetic dependencies, drug-sensitizing genes, or resistance modifiers at the genome scale, including determinants of PARP/ATR inhibitor response. In contrast, HRD or BRCA1/2-mutant models may poorly tolerate constitutive Cas9 activity because Cas9-induced double-strand breaks can trigger excess DNA damage; in these settings, transient Cas9 ribonucleoprotein delivery, inducible Cas9 systems, or lower-complexity arrayed editing approaches may reduce toxicity and selection artifacts. CRISPRi and CRISPRa are particularly useful for essential genes, dosage-sensitive pathways, enhancer/regulatory elements, and genes where complete knockout is lethal or biologically misleading, because they allow graded repression or activation without introducing double-strand breaks. Base editing and prime editing provide complementary strategies for modeling clinically relevant point mutations, splice-site changes, drug-resistance alleles, or BRCA1/2 reversion events while minimizing double-strand break–associated toxicity. Finally, CRISPR-edited organoids and GEMMs provide more physiologic platforms for studying tumor initiation, clonal evolution, cell-of-origin biology, and therapy resistance in tissue context, as demonstrated by CRISPR-modified fallopian tube/oviductal and ovarian surface epithelial organoid models and CRISPR-engineered murine HGSC models (7, 64). Thus, CRISPR-based studies in HGSC should explicitly report the rationale for the editing modality, the DNA repair background of the model, the delivery approach, the passage number, the editing efficiency, the validation strategy, and the potential selection pressures introduced by the editing system.

### Maintenance of cell lines

In addition to understanding the choice and optimization of ovarian cancer cell lines, it is imperative to consider proper care and the limitations of certain cell lines. Regularly profiling cell lines, using low-passage cells (i.e., fewer than 20 passages), and monthly testing for *mycoplasma* are essential steps for conducting rigorous experimentation; specific guidelines and recommendations can be found in previously published work (65). The methodology used in the lab to conduct these steps should be reported in all manuscripts and grant applications, thereby reducing the risk of using contaminated cell lines. In addition to these standard practices, researchers should also consider and report the ethnicity and age of the patient from whom the cells were isolated. Single-nucleotide polymorphisms (SNPs) vary across ethnicities, and although most SNPs are nonfunctional, some functional SNPs confer a selective advantage (66). The prognosis of older patients tends to be worse compared to that of younger individuals with a similar diagnosis. Notably, while there are likely additional co-morbidities in older patients, the poorer prognosis cannot be fully disentangled from a potentially more aggressive disease (67, 68). As with all research fields, the more we understand the underlying biology of HGSC, the more we can identify experimental models that may not fully represent the disease of interest.

### Limitations of cell lines

The primary limitation of any cell line lies in the risk of cell lines that are not definitively of a given origin. This risk can be mitigated by verifying the molecular profile as described above and using the HGSC cell lines listed in Table 1. In addition to this limitation to specific cell lines, cell lines run the risk of drift, both histologically and molecularly. For instance, a systematic approach to identifying and authenticating gynecologic cancer cell lines identified several cell lines that had been misidentified, redundant, or cross-contaminated with other lines (69). To mitigate this risk, cell line sourcing should be documented, and “STR” (Short Tandem Repeat) profiling should be performed regularly. Finally, working with cell lines as a primary model is limited, given that the cells are removed from their tumor microenvironment and often cultured in 2D on tissue culture plastic with nutrient-rich media. Thus, the use of 3D cultures with an extracellular matrix and physiological culture media (e.g., plasma-like media) is a technical approach to better recapitulate physiological conditions (70, 71).

## Ovarian cancer spheroids and organoids

### Spheroids

Transcoelomic metastasis, the most common route of metastasis in ovarian cancer, contributes significantly to morbidity and mortality in women with HGSC (72), due to the detrimental effects on important organs within the abdomen, including the peritoneum, the omentum, the bladder, the liver, the lungs, and the intestines (72). In addition, malignant ascites often results from transcoelomic metastases, in which HGSC cells detach from the primary tumor and shed into the peritoneal cavity (73). However, instead of remaining as single cells, HGSC cells organize into free-floating 3D multicellular aggregates called spheroids (73), which enables HGSC to evade anoikis (74), a type of programmed cell death triggered when epithelial cells detach from the extracellular matrix (75). Spheroids are highly heterogeneous, containing HGSC cells and stromal cells (TAMs, CAFs, mesothelial cells) (76). The complex 3D structure of spheroids also contributes to immune evasion (77). Spheroids adhere to the mesothelium, a single-cell layer lining the peritoneal cavity that acts as a physical barrier against tumor implantation. Upon contact, HGSC cells facilitate mesothelial clearance, allowing cancer cells to anchor directly to the underlying submesothelial matrix and establish secondary tumors (78). Ovarian multicellular spheroids from malignant effusion specimens have been developed and used to test chemoresponses (79). In addition, spheroids derived from malignant ascites have been used to establish PDX models and tested in a humanized mouse model of orthotopic ovarian cancer (80). Furthermore, 3D models have been developed using ovarian cancer spheroids as model systems for cells undergoing metastasis as well as for chemotherapeutic testing (81, 82). Overall, spheroids derived from malignant ascites provide clinically relevant models to examine cancer cell/immune cell interactions and test anticancer therapies, including immune checkpoint inhibitors and other immunomodulatory agents (83–85).

### Patient-derived organoids (PDOs)

Despite being valuable resources, HGSC cell lines and spheroid models often differ in their genetic makeup and fail to fully capture the heterogeneity of the patient tumors from which they were derived, thereby limiting their utility (21). More sophisticated models, such as patient-derived organoids (PDOs), have the potential to address these limitations and provide a system that more faithfully recapitulates tumor complexity and therapeutic response. Tamura et al. (86) demonstrated the feasibility of generating patient-derived organoids for several tumor types, including ovarian cancer, using ultralow attachment plates. However, a breakthrough came with the successful establishment of short-term organoid cultures from tumor biopsies of 22 HGSC patients and one low-grade serous carcinoma patient (87). Thirty-four organoid lines were generated from these 23 patients, using cells derived from primary, metastatic, or recurrent tumor sites. These organoids retained the histological features and molecular profiles of the original tumors, including PAX8 expression and *TP53* mutations. HRD organoids exhibited sensitivity to PARP inhibitors, highlighting the utility of these organoid models for personalized treatment strategies (87).

Subsequently, several studies across different ovarian cancer histologic subtypes have used organoid models to elucidate factors that influence both disease progression and therapeutic response (Table 3; Supplementary Table S2). Kopper et al. established 56 organoid lines from 32 patients with ovarian cancer, representing all major histotypes, and demonstrated their ability to retain intra- and interpatient heterogeneity upon long-term culture (88). Interestingly, most single-nucleotide variants and structural variants present in the original tumor were also preserved in the organoids, even after extended passaging. These findings were further substantiated by other studies (89, 90).

**TABLE 3:** Summary of ovarian cancer organoids.

Study	Organoid model(s)	Summary of results
Kopper et al 2022 (88)	56 organoid lines from 32 patients (23 high-grade serous carcinoma)	Retained PAX8 expression, mutated p53 expression, and copy number variations Differing drug sensitivities
Maenhoudt et al 2020 (90)	22 high-grade serous carcinoma organoids (from dissociated biopsies)	Improved culture medium (neuregulin 1) Retained p53 and BRCA mutations, copy number alterations, and PAX8 and hormone receptor expression
de Witte et al 2020 (89)	13 high-grade serous carcinoma patient-derived organoids were developed	67% of single nucleotide variants overlap with organoid and primary tumors, with 27% exclusive to the patient-derived organoid
Velletri et al 2022 (93)	High-grade serous carcinoma ascites (5 patients)	Derived from single-cell samples and patient-derived ascitic fluid to create 3D cultures Maintained patient-specific features, cancer-initiating properties, and resistance to cell death Enrichment for cancer stemness pathways, drug resistance, hypoxia, JAK/STAT3 signaling, and epithelial-mesencyhmal transition
Nanki et al 2020 (92)	7 organoids were created, including 3 high-grade serous carcinoma cases	Established a protocol to culture and expand primary tumors using a specialized Matrigel-based system A 90% success take rate, and 3 derived lines All retained histological and genetic characteristics of the primary tumors (chromosomal aberrations)

### Organoid culture medium and methodologies

Maenhoudt et al. successfully established organoid models derived from ovarian cancer tissues that closely mimicked patient-specific tumor characteristics and optimized culture conditions, thereby developing an enhanced organoid culture medium (OCOM4) that improved organoid formation efficiency (90). Notably, MUC16/CA125, widely used as a biomarker for advanced-stage ovarian cancer (91), was consistently expressed in organoids. Nanki et al. established a protocol for culturing and expanding single cells dissociated from primary ovarian tumors using a specialized Matrigel-based system with niche factor cocktails. This method successfully generated organoids from multiple ovarian cancer subtypes, including HGSC, endometrioid carcinoma, and clear cell carcinoma, with an 80% success rate (92). These organoids retained the histological characteristics and genetic profiles of the primary tumors, including key cancer-related mutations (e.g., *BRCA1*, *BRCA2*, *TP53*, and *PIK3CA*) and most chromosomal aberrations present in the original tumors. Beyond their genetic and phenotypic fidelity, epithelial ovarian cancer-derived organoids also demonstrated utility as *in vitro* platforms for drug screening. When tested against commonly used chemotherapeutic agents, including paclitaxel, carboplatin, doxorubicin, and gemcitabine, organoid responses closely mirrored those of the patient tumors, indicating the potential of organoids to predict tumor-specific drug sensitivities and guide precision medicine strategies (90).

Given the pivotal role that multicellular aggregates in ascites play in ovarian cancer dissemination, developing models that preserve their physiological and pathological features is critical. Velletri et al. (93) introduced a novel approach to generate single-cell-derived metastatic ovarian cancer spheroids from patient-derived ascitic fluid. By isolating single cells from patients with HGSC and culturing them under 3D conditions supplemented with ascitic fluid, they created spheroids that closely resemble *in vivo* tumor conditions. Single-cell RNA sequencing provided a comprehensive characterization of these spheroids, revealing key subpopulations that were maintained or enriched in single-cell-derived metastatic ovarian cancer spheroids. These spheroids retained patient-specific features, cancer-initiating properties, and resistance to cell death, making them valuable tools for studying metastasis and testing personalized therapies.

### Advantages of organoids

By bridging the gap between heavily selected immortalized cell lines and heterogeneous tumor samples, organoid models have had a significant impact on ovarian cancer research. These models successfully capture the genetic and histopathological features of primary tumors, making them invaluable for studying tumor biology, intratumoral heterogeneity, and treatment responses. The ability to predict treatment responses using organoid-based drug sensitivity and resistance testing may enhance personalized therapeutic strategies, improving clinical outcomes for ovarian cancer patients. Furthermore, the identification of key genetic alterations, such as HRD-like copy number variations and platinum/PARP inhibitor sensitivity, would allow for patient classification based on tumor genomics, enabling more precise treatment selection. By incorporating these models into real-time drug screening, oncologists can tailor treatment plans to individual tumor profiles, thereby minimizing the use of ineffective therapies and reducing adverse effects. Additionally, these organoids provide a powerful platform for discovering novel therapeutic targets and testing innovative combination therapies, ultimately advancing ovarian cancer management and improving patient survival. Future implementation of organoids in ovarian cancer research will also be critical to align with recent NIH/NCI guidelines related to *in vivo* models.

### Limitations of organoids as ovarian cancer models

Because only certain cell populations in the tumor successfully adapt and expand under the *in vitro* conditions used to generate organoids, the resulting organoids may not capture the biological diversity of the original tumor. This can create selection bias in the patient-derived organoid models and is an important limitation to consider (94). Although PDOs may not capture the full spectrum of dynamic clonal evolution observed in patients, they can model adaptive responses to therapeutic pressure. Several studies have demonstrated that sustained exposure to chemotherapeutic agents or targeted therapies can generate resistant organoid models that recapitulate key features of acquired resistance, including transcriptional reprogramming, altered signaling pathways, and drug-tolerant persister states (88, 95, 96). These findings highlight the utility of PDOs as a platform for studying therapy-induced adaptation and resistance mechanisms in a controlled, patient-specific context. However, these adaptations occur in the absence of systemic influences, including immune interactions and pharmacokinetic constraints, and therefore are best interpreted alongside complementary *in vivo* models.

A limitation of organoids is the incomplete representation of the TME, including immune, stromal, and vascular interactions. However, recent advances in co-culture methodologies have begun to address this limitation. For example, multi-component culture systems incorporating tumor cells alongside fibroblasts, endothelial cells, and immune populations have demonstrated improved modeling of tumor–stromal crosstalk, immunosuppression, and therapeutic response. Notably, recent work by Balkwill and colleagues describes a “pentaculture” system integrating ovarian cancer cells with mesothelial cells, fibroblasts, adipocytes, and macrophages, enabling interrogation of key microenvironmental interactions relevant to omental metastasis and immune modulation (97). Additional studies incorporating tumor-infiltrating myeloid cells (e.g., macrophages) and lymphocytes (e.g., T cells) into organoid or spheroid systems further demonstrate the feasibility of modeling immune–tumor interactions and responses to immunotherapy *in vitro* (83, 98). In addition, the clonal selection of each cell type within organoids can be affected by the medium or selection pressure used, variations in the composition of Matrigel or basement membrane extract, and the number of passages. These can lead to reproducibility issues when using these models (94). Consistently, CAFs, macrophages, and T-cells can exist in different states, and their impact on tumor progression and therapeutic response is state-dependent. The organoid models may fail to capture these complexities (94). While these approaches do not fully recapitulate the systemic and spatial complexity of *in vivo* tumors, they represent an important step toward more physiologically relevant *in vitro* models and provide a tractable platform to dissect defined cellular interactions within the HGSC tumor microenvironment.

## ORGAN-ON-A-CHIP

While organoids help overcome some limitations of 2D cell culture models and better capture the genetic and pathological features of tumor tissues, they still do not model the complex dynamic characteristics of tumor tissues. In addition to cancer cells, tumors are composed of several cell types, such as stromal and immune cells, within a microenvironment characterized by a dense extracellular matrix, irregular blood vessels, and variable concentrations of nutrients and oxygen. While 3D models can mimic some cell-cell and cell-extracellular matrix interactions and incorporate biochemical gradients, they fail to accurately model more complex tumor microenvironment interactions such as vascularization and other mechanical cues (e.g., blood flow). Organ-on-a-chip technology can address these limitations. These microfabricated devices enable the co-culture of tumor cells and stromal cells with relevant extracellular matrix components and can combine these with the advantages of microfluidic technology to mimic the complex biochemical gradients observed in tumor tissues. This approach is particularly relevant when considering biological questions such as tumor-cell migration, ascites-driven shear stress or vascular barrier interactions (99–101). Whereas spheroid or organoid models may be sufficient for questions limited to intrinsic tumor cell viability or drug sensitivity.

Many organ-on-a-chip models of ovarian cancer have been developed so far. Rizvi et al. developed a customizable microfluidic platform that uses cell line-derived 3D ovarian cancer spheroids to investigate the role of fluid forces in transcoelomic ovarian cancer metastasis via ascites (102). Using this model, they show that fluid flow induces epithelial-mesenchymal transition and promotes an aggressive tumor phenotype. Others have described a low-cost, easy-to-use organ-on-a-chip model for studying ovarian cancer cell migration (103). More complex microfluidics-based platforms have demonstrated that chemokine- and folic acid-loaded nanoparticles diffuse across blood vessels and home to ovarian cancer cells that express folate receptors, ultimately inducing the migration of dendritic cells and T-cells toward these cells (104). Other organ-on-a-chip models include platforms that have served as valuable tools for studying platelet extravasation (105, 106), tumor-neutrophil dynamics (107), and metastasis of ovarian cancer cells to the omentum (108). Furthermore, in addition to modeling complex cellular, biochemical, and biophysical interactions within tumor tissues, microfluidic systems can be used to link different organs-on-a-chip, potentially simulating the complexity of the multiorgan system. Such models could be useful for evaluating metabolism (drug or nutrient) and pharmacokinetics. Therefore, by offering the unique ability to incorporate several nuances of complex *in vivo* systems, the organ-on-a-chip models have served as a much-needed bridge between traditional *in vitro* and *in vivo* ovarian cancer models.

### Limitations of organ-on-a-chip

While undoubtedly powerful, these models can be constrained by the availability of primary human tissue and by bioengineering expertise and equipment. On a related note, these limitations also affect the scalability and reproducibility of organ-on-a-chip technology. Organs-on-a-chip are powerful tools given the complexity of multiple cell types and the environment, but this is also a limitation because the combined cell types are often derived from different patients, potentially introducing biological confounders such as HLA mismatches, patient ancestry, and patient age. For instance, an elegant study using an organ-on-a-chip investigated the role of platelets in HGSC cell metastasis; however, the investigators used cells from four different sources and backgrounds, potentially affecting the results (106). The development of “autologous” organ-on-a-chip models, which use patient-derived materials from a single patient, will help overcome these limitations, realize their full potential, and help organs-on-a-chip become a mainstream platform for both basic and translational research.

## Tumor slices

Tumor slice cultures represent a highly translational *ex vivo* platform that preserves native tumor architecture, multicellular composition, and spatial relationships within HGSC. Unlike dissociated systems, tumor slices maintain tumor–stromal–immune interactions and are particularly well-suited for evaluating spatial pharmacodynamics and short-term therapeutic responses (109–111). Tumor slices can be generated using precision instruments such as Krumdieck tissue slicers, producing uniform sections (commonly ∼250–400 μm thick) that balance structural integrity with adequate oxygen and nutrient diffusion. Optimal slice thickness is critical for maintaining tissue viability while minimizing diffusion limitations. Most studies use slices of 250–350 μm thickness, as thicker sections (>400 μm) are prone to central hypoxia and necrosis, whereas thinner sections may compromise structural fidelity (112). Under optimized culture conditions, tumor slices remain viable for approximately 48–96 h, with some reports extending functional readouts up to 5–7 days depending on media composition, oxygenation, and tissue quality (110, 112). Viability is typically assessed using ATP-based assays, live/dead staining, or preservation of proliferative (Ki67) and apoptotic (cleaved caspase-3) markers.

Necrosis represents a key technical challenge in tumor slice cultures. To mitigate this, investigators often exclude visibly necrotic tumor regions during sampling, utilize dynamic culture systems such as air–liquid interface platforms or perfusion-based systems to improve oxygenation, and normalize experimental design by using multiple slices per condition to account for intra-tumoral variability. Histologic pre-screening, while challenging, and post-culture validation are important to ensure that observed treatment responses are not confounded by tissue necrosis.

A major advantage of tumor slices is the retention of endogenous immune populations, including tumor-associated macrophages, T cells, and other stromal components (111). These systems enable short-term interrogation of immune activation states, cytokine signaling, and cell–cell interactions within an intact tumor microenvironment. However, immune cell viability and functionality decline over time in culture, necessitating early time-point analyses (typically within 24–72 h). Importantly, because these systems lack vascular input, they do not support active recruitment of immune cells, limiting their ability to model dynamic immune trafficking.

Tumor slices are uniquely suited for high-dimensional spatial analyses. Multiplexed immunohistochemistry, imaging mass cytometry, and spatial transcriptomics can be applied to treated and untreated slices to assess drug-induced changes in tumor and microenvironmental compartments. These approaches enable quantification of spatial relationships such as immune cell proximity, tumor–stroma interactions, and regional heterogeneity in signaling pathways. For example, prior studies have combined tumor slice cultures with multispectral imaging to assess cytotoxic T-cell activation (e.g., granzyme B) (111), DNA damage responses, and immune checkpoint expression following therapeutic intervention.

Tumor slice assays are particularly powerful for measuring pharmacodynamic responses in a clinically relevant context. Common endpoints include apoptosis, proliferation, DNA damage (γH2AX), pathway inhibition, and immune modulation (e.g., PD-L1 expression, cytokine production) (110, 111). Because slices preserve spatial heterogeneity, they enable evaluation of region-specific drug penetration and response, providing insights into therapeutic resistance mechanisms not captured in homogeneous culture systems.

To enhance reproducibility and enable broader adoption, there is a growing need for standardized protocols across laboratories (112, 113). Key parameters include slice thickness, time from surgical resection to culture, media composition, oxygenation method (static vs. perfusion), and endpoint assays. Implementation of quality-control metrics, such as baseline viability thresholds, histopathologic validation, and inclusion of technical replicates, will be essential for cross-study comparisons. Integration of tumor slice assays into preclinical pipelines may also benefit from alignment with clinical trial timelines, particularly for window-of-opportunity studies.

### Limitations of tumor slices

Despite their translational advantages, tumor slice models have several important limitations. First, these systems are inherently short-lived, with most experiments constrained to ≤96 h due to declining cellular viability and immune function. Second, the absence of vascular perfusion restricts nutrient delivery, drug distribution, and immune-cell trafficking, limiting the ability to model dynamic systemic interactions. Third, tumor slices are typically derived from a single spatial region of a tumor and therefore may not fully capture the extensive intra-patient heterogeneity characteristic of HGSC, particularly across metastatic sites and ascites-derived disease.

Additionally, variability in tissue quality, degree of necrosis, and surgical handling introduces experimental heterogeneity. The finite amount of tissue available necessitates careful allocation of slices across experimental conditions, often requiring multiple slices per treatment group to ensure statistical robustness. Finally, while tumor slices preserve endogenous immune populations, they lack the capacity to model immune recruitment or long-term immune remodeling and thus are best positioned as short-term platforms for evaluating spatial pharmacodynamics rather than longitudinal tumor evolution.

## Emerging *IN VIVO* models of high-grade serous carcinoma

Organoids and cell lines that genomically resemble HGSC have been extensively evaluated for growth characteristics in murine xenografts. Historically, cell line xenograft models have been the primary approach for assessing *in vivo* cancer biology; however, HGSC cell lines exhibit lower-than-expected tumor take rates in immunocompromised mice. This also highlights the limitation of evaluating non-tumor cell interactions (i.e., stromal and immune cells) within the tumor microenvironment in cell line xenograft models. The importance of the stromal and immune tumor microenvironment in HGSC progression, metastasis, and therapeutic response has motivated the development of models that incorporate this context. For instance, *in vivo* models have evolved from cell line xenografts to patient-derived xenografts (PDX) grown in humanized murine models or syngeneic models. Recent atlas-level studies provide important reference frameworks for understanding the HGSC tumor microenvironment. A multimodal spatial pre-cancer atlas of fallopian tube precursors demonstrates immune suppression and microenvironmental remodeling during progression toward HGSC, providing a resource for mapping early stromal and immune changes in p53 signatures/STIC-associated lesions (6). Complementary HGSC scRNA-seq atlases define the cellular architecture of established disease, including malignant epithelial states, CAF populations, macrophage/myeloid subsets, T-cell exhaustion programs, B/plasma-cell niches, and metastatic-site-specific immune remodeling (114–116). Note, most of these studies can be interrogated through the Chan Zuckerberg CELL by Gene Discovery platform (https://cellxgene.cziscience.com/). These studies should be used as reference datasets when selecting models to interrogate specific TME compartments. Accordingly, this section will discuss more recently developed *in vivo* models that attempt to capture the biological role of the complex HGSC tumor microenvironment.

### Patient-derived xenograft (PDX) and humanized stroma PDX models

Cancer cell line xenografts are commonly used for *in vivo* studies due to the ease of propagation and rapid growth. Interestingly, most HGSC cell lines fail to form robust tumors and recapitulate the tumor microenvironment *in vivo*, due to several factors, including (i) lack of significant cellular heterogeneity (compared to primary patient tumors), (ii) little-to-no tumor stroma, and the stroma that is present is murine, not human, and (iii) by necessity, human cell xenografts are generated in immunosuppressed mice, resulting in models that lack competent immune systems (38). Overcoming these limitations is necessary because researchers continue to define the underlying contribution of non-cancer cells (e.g., fibroblasts, macrophages) to disease progression and therapeutic response.

To address some of these limitations, human PDX tumor models have been developed to better reflect the heterogeneity of human tumors and improve therapeutic testing by creating a potential “avatar” of a patient’s tumor in a mouse. PDX, while laborious to develop, reflects the genomics of the primary tumor, the activation of expected tumor drivers and DNA repair pathways, and the secretion of CA125 (117, 118). Like organoids, PDXs can be used to study responses to standard chemotherapies. While PDX models may be an improvement over cell line xenografts, they still have limitations. PDXs are costly and challenging to develop because they require access to fresh primary tissues. Engraftment rates can be as low as 30%–50% (119), suggesting that the murine host is not ideal for supporting human cancer growth. Disappointingly, with the selection pressures of engraftment and selection for rapid growth, Clonal selection of dominant aggressive subclones occurs early in the course of PDX engraftment such that PDXs lose intratumoral heterogeneity with serial passaging (120, 121). As such, after passage 5, when the PDX has expanded enough for larger-scale studies, it has lost much of the heterogeneity that initially made it attractive as a tool for study. Concurrent with the clonal evolution of the PDX with passaging, there is a loss of human stroma. While human tumor stroma can be present with the primary implanted tumor and early passages, human tumor stromal cells are typically lost by passage 3, resulting in exclusively murine stroma after preparation. With the loss of human tumor stromal cells, the human extracellular matrix (ECM) is also lost, altering signaling cues and cell-matrix interactions (122). Combined with the loss of human stroma/ECM, clonal selection results in genomic and transcriptional drift, which can alter therapeutic response (121). Furthermore, as in cell line xenografts, traditional PDX must be engrafted in immunosuppressed mice. Thus, given the lack of human stromal and immune cells, PDX cannot fully recapitulate the patient’s tumor.

### Humanized immune systems

Due to these issues with PDX, significant efforts have been made to use human hematopoietic stem cells from various sources to generate mice with human immune systems (123). PDX can engraft and grow in these humanized mice, even in the setting of an allogeneic immune system. Furthermore, immune cells can infiltrate tumors and impact cancer cell expression profiles, resulting in xenografts that more closely resemble primary tumor expression profiles (124).

For ovarian cancer-specific models, Gitto and colleagues orthotopically engrafted a PDX into a mouse and administered tumor-infiltrating lymphocytes derived from the same patient’s tumors (125, 126). This model has the advantage of using autologous tumor/T cells, providing a platform for studying human tumor-infiltrating lymphocytes *in vivo*. However, the model lacks other human immune cells (i.e., TAMs, NKs) and human stromal cells, is limited by the availability of patient-matched immune cells and can eventually develop graft-versus-host disease. More recently, Steinkamp and colleagues engrafted human ovarian PDX models derived from ascites into NBSGW (NOD.Cg-Kit^W-41J^ Tyr^+^ Prkdc^scid^ Il2rg^tm1Wjl/ThomJ^) mice in which the marrow was humanized with human CD34^+^ hematopoietic stem cells (80). This strain, on an NSG background, carries a *c-kit* mutation. The *c-kit* mutation reduces the ability of murine hematopoietic stem cells to engraft, allowing increased engraftment of transplanted human CD34^+^ hematopoietic stem cells. This model had robust myeloid cell engraftment, which is sometimes lacking in other humanized bone marrow mouse models. While this has a “fully” human immune compartment, it is allogeneic, as the transplanted immune system does not come from the same patient as the PDX. Furthermore, this model still lacks human stroma.

### Humanized stromal models

Human tumor stromal cells (fibroblasts, adipocytes, endothelial cells) play critical roles in promoting tumor growth and metastasis and in regulating the immune compartment (127–129). Therefore, incorporating human stroma into tumors is vital in understanding the complex tumor microenvironment. To address this issue, Yang and colleagues combined (i) patient-derived tumor cells, (ii) patient-derived cancer-associated mesenchymal stem/progenitor cells, and (iii) human endothelial cells. These cells were then engrafted into mice with humanized bone marrow to create a humanized immune stroma PDX (HIS-PDX) model (130). RNA-sequencing analysis of the HIS-PDX model with human connective tissues, vasculature, and immune cell infiltrates demonstrated a 94%–96% correlation with the primary human tumor. As with patients with HGSC tumors, the model proved resistant to immune checkpoint inhibitor therapy, offering an opportunity to better understand immunotherapy resistance in human tumors. However, like other models, this model has the weakness of having an allogeneic immune compartment. Also, contributing to the complexity of adding non-tumor cell types to HIS-PDX models, scRNA-seq technology has revealed emerging evidence of different fibroblast types that can have functionally distinct roles in supporting the tumor (131). Ultimately, finding ways to better engraft patient peripheral blood monocyte-derived hematopoietic cells could enable an autologous immune system and a completely humanized mouse model of ovarian cancer.

### Limitations of PDX and humanized PDX models

Humanized PDX models provide an important platform for evaluating human tumor–immune interactions, but they should not be interpreted as fully immune-competent human systems. CD34^+^ hematopoietic stem cell–humanized mice often show incomplete immune reconstitution, with variable T-cell education, limited B-cell maturation, impaired lymphoid architecture, and underdeveloped myeloid and NK-cell compartments, particularly in strains lacking human cytokine support (132, 133). Species-specific cytokine incompatibility further limits the differentiation, survival, and function of human innate immune cells; newer models incorporating human cytokines or knock-in alleles improve myeloid and NK-cell development but do not fully normalize immune function (80, 134, 135). In PBMC-humanized models, rapid T-cell engraftment enables short-term immunotherapy testing, but xenogeneic graft-versus-host disease frequently restricts experimental duration and can confound interpretation of anti-tumor immune activity. A further limitation is HLA mismatch; in many humanized PDX studies, the tumor, hematopoietic donor, and stromal components are not autologous, creating an allogeneic immune context that may alter antigen recognition, T-cell activation, immune editing, and response to checkpoint blockade. Thus, while humanized models are valuable for translational immuno-oncology, results should be interpreted with attention to donor matching, immune reconstitution quality, cytokine support, graft-versus-host disease risk, and whether the model uses autologous versus allogeneic immune components.

### Syngeneic models

Syngeneic tumor models are generated by engrafting transformed cells from mouse tissues into another immunocompetent mouse of the same strain. By ensuring that the genetic and immunologic compatibility is preserved between the implanted cancer cells and the host mice, graft-versus-host rejection is avoided, which is critical for successful engrafting of cancer cells in an immune-competent host. The most significant advantage of these models over xenograft models is their utility for studying tumor progression and therapeutic efficacy in a fully functional host immune system. Being able to do so is vital for understanding the interactions between tumor cells and host stromal and immune cells, as well as for determining how these interactions shape the tumor microenvironment. These models also provide a robust, rapid, and reproducible platform to test new immunotherapeutic strategies. Given their undeniable value, several murine ovarian cancer cell lines have been successfully derived and used to develop syngeneic models. These cell lines differ in the strain of mice from which they were derived (C57BL6, FVB, or mixed background), their site of origin (ovarian surface versus fallopian tube epithelium), and the method used to generate them. While some cells were derived directly from ovarian tumors or ascites that developed in transgenic mice, others were generated by transforming primary murine fallopian tube or ovarian surface epithelial cells in culture. *In vitro* transformation of fallopian tube or ovarian surface epithelial cells was achieved either through spontaneous accumulation of genetic changes during repeated passaging or by engineering specific clinically important genomic alterations. Table 4 lists commonly used mouse ovarian cancer cell lines used to develop syngeneic mouse models and summarizes their similarities and differences.

**TABLE 4:** List of murine ovarian cancer cell lines commonly used to generate syngeneic mouse models of high-grade serous carcinoma. Note that this is not an exhaustive list of all murine ovarian cancer cell lines.

Cell line	Genetic alterations	Mouse strain	Origin	Histology	Reference
ID8	Spontaneous transformation in culture	C57BL/6/Mixed	Ovarian surface epithelium	Serous	Roby KF et al., carcinogenesis. 2000 (136).
BR5-FVB1	p53^−/−^, Myc^OE^, K-ras^G12D^	FVB	Ovarian surface epithelium derived BR5 cells were injected i.p into FVB mice and derived from i.p tumors that were formed	Serous	Xing D and Orsulic S. Proc Natl Acad Sci United States 2005 (160).
BR (BR5FVB1 + Akt)	p53^−/−^, Myc^OE^, K-ras^G12D^, Akt^OE^	FVB	Ovarian surface epithelium (BR5-FVB1 cells were infected with RCAS-Akt)	Serous	Xing D and Orsulic S. Proc Natl Acad Sci United States 2005 (160).
MOVCAR	Ascites of tumor-bearing C57BL/6 TgMISIIR-TAg transgenic mice	C57BL/6	Ascites (primary tumor site could be ovarian surface epithelium, fallopian tube epithelium, or uterus)	Serous	Quinn BA et al., J ovarian res. 2010 (161).
STOSE	Spontaneous transformation in culture	FVB/N	Ovarian surface epithelium	Serous	McCloskey CW at al., Front Oncol. 2014 (162).
30200	p53^−/−^, BRCA1^−/−^, Tag_121_^OE^ (Rb inactivation)	FVB	Ovarian surface epithelium	Serous	Szabova L et al., 2014 (163)
60577	p53^−/−^, BRCA1^−/−^, Tag_121_^OE^ (Rb inactivation)	FVB	Ovarian surface epithelium	Serous	Szabova L et al., 2014 (163)
ID8-Trp53^−/−^	p53^−/−^	C57BL/6	Ovarian surface epithelium	Serous	Walton J et al., cancer res. 2016 (138)
ID8-Trp53^−/−^, Brca1^−/−^	p53^−/−^, Brca1^−/−^	C57BL/6	Ovarian surface epithelium	Serous	Walton J et al., Sci Rep. 2017 (164).
ID8-Trp53^−/−^, Brca2^−/−^	p53^−/−^, Brca2^−/−^	C57BL/6	Ovarian surface epithelium	Serous	Walton J et al., cancer res. 2016 (138).
ID8-Trp53^−/−^, Nf1^−/−^	p53^−/−^, Nf1^−/−^	C57BL/6	Ovarian surface epithelium	Serous	Walton J et al., Sci Rep. 2017 (164).
ID8- Trp53^−/−^, Nf1^−/−^	p53^−/−^, Nf1^−/−^	C57BL/6	Ovarian surface epithelium	Serous	Walton J et al., Sci Rep. 2017 (164).
ID8-Trp53^−/−^, BRCA2^−/−^, Pten^−/−^	p53^−/−^, BRCA2^−/−^, Pten^−/−^	C57BL/6	Ovarian surface epithelium	Serous	Walton J et al., Sci Rep. 2017 (164)
HGS1	p53^−/−^, BRCA2^−/−^, Pten^−/−^	C57BL/6	Orthotopic from peritoneal tumor (originally the cells were derived from transformed fallopian tube epithelium in a GEMM)	Serous	Maniati E et al., cell reports. 2020 (64).
HGS2	p53^−/−^, BRCA2^−/−^, Pten^−/−^	C57BL/6	Orthotopic from ovarian/fallopian tube tumor (originally the cells were derived from transformed fallopian tube epithelium in a GEMM)	Serous	Maniati E et al., cell reports. 2020 (64).
HGS3	p53^−/−^, BRCA2^−/−^, Pten^−/−^	C57BL/6	Orthotopic from fallopian tube tumor (originally the cells were derived from transformed fallopian tube epithelium in a GEMM)	Serous	Maniati E et al., cell reports. 2020 (64).
HGS4	p53^−/−^, BRCA2^−/−^, Pten^−/−^	C57BL/6	Orthotopic from mesenteric tumor (originally the cells were derived from transformed fallopian tube epithelium in a GEMM)	Serous	Maniati E et al., cell reports. 2020 (64).
BPCA	Brd4^OE^, p53^R172H^, CCNE1^OE^, Akt2^OE^	C57BL/6	Fallopian tube epithelium	Serous	Iyer S et al., cancer Discov. 2021 (140).
SPCA	Smarca44^OE^, p53^R172H^, CCNE1^OE^, Akt2^OE^	C57BL/6	Fallopian tube epithelium	Serous	Iyer S et al., cancer Discov. 2021 (140).
KPCA	KRAS^G12V^, p53^R172H^, CCNE1^OE^, Akt2^OE^	C57BL/6	Fallopian tube epithelium	Serous	Iyer S et al., cancer Discov. 2021 (140).
PPNM	p53^R172H^, Pten^−/−^, Nf1^−/−^, MYC^OE^	C57BL/6	Fallopian tube epithelium	Serous	Iyer S et al., cancer Discov. 2021 (140).
BPPNM	Brca1^−/−^, p53^R172H^, Pten^−/−^ Nf1^−/−^ MYC^OE^	C57BL/6	Fallopian tube epithelium	Serous	Iyer S et al., cancer Discov. 2021 (140).
SO	p53^−/−^ CCNE1^OE^ MYC^OE^ HRAS^G12V^	C57BL/6	Ovarian surface epithelium	Serous	Agadjanian et al., Sci. Rep. 2025 (165).

### Murine ID8 model

An important consideration when choosing cell lines for developing syngeneic models is the ovarian cancer subtype being modeled. For example, the murine ID8 cell lines have served as workhorses for developing syngeneic mouse models of ovarian cancer (136, 137). These cells were derived through spontaneous transformation of ovarian surface epithelial cells from C57BL6 mice in culture (136). Although they reliably develop serous tumors when injected either intraperitoneally or intrabursally in syngeneic C57BL6 mice, the tumors formed by the parental ID8 cells do not reflect the molecular features of human HGSC tumors (138). To address this limitation in ID8 cells, Walton et al. genetically modified the parental ID8 line to generate Trp53-and BRCA1/2-null versions that more closely align with human disease (138). It is worth noting that, despite sharing the same origin, two cell lines can differ from each other. For instance, in one study in which ID8 and STOSE cells were both derived from ovarian surface epithelial cells via spontaneous transformation, they exhibited differences at the molecular and phenotypic levels (139), likely due to differences in the mouse strain used and distinct genomic alterations that led to their transformation. Therefore, such differences between the models should be considered when interpreting the results.

### Murine fallopian tube epithelium model

As understanding of the origin of HGSC shifts from the ovarian surface epithelium towards fallopian tube epithelial cells, syngeneic murine cell lines that reflect this paradigm shift need to be established. In response to this need, several groups have introduced genetic alterations in murine fallopian tube epithelial cells that reflect those observed in human cancers. For example, Iyer et al. engineered mutant fallopian tube epithelial cells derived from Trp53^−/−^ and Trp53−/−Brca1−/− mice to establish several genetically defined murine HGSC cell lines that formed serous tumors (140). These cell lines mirror both the suspected cell of origin and key driver mutations seen in patients with HGSC.

Despite syngeneic models being powerful resources for understanding disease etiology and testing new treatment strategies, these models are often cultured *in vitro* before implantation and are therefore subject to the same limitations noted in the cell line section. Consequently, given HGSC disease heterogeneity, research conclusions about HGSC biology should not be based entirely on non-HGSC-conforming cell lines; the findings should be complemented by additional cell lines, murine models, or primary human tumors.

Syngeneic models provide a critical platform for studying tumor–immune interactions within an intact, fully functional host immune system. Because tumor cells are implanted into immunocompetent mice of the same genetic background, these models enable mechanistic interrogation of anti-tumor immunity, including T-cell priming, myeloid cell recruitment, immune checkpoint signaling, and response to immunotherapies such as checkpoint blockade or combination immune-modulating strategies (141–144). This immunologic fidelity represents a major advantage over xenograft and humanized systems, particularly for studying dynamic immune responses and host–tumor co-evolution.

### Limitations of syngeneic models

The strength in immunologic modeling is offset by limited fidelity to human HGSC. Most commonly used syngeneic ovarian cancer models, such as ID8 and its derivatives, originate from murine ovarian surface epithelium and do not fully recapitulate the molecular hallmarks of HGSC, including the near-universal TP53 mutation, widespread chromosomal instability, and the genomic complexity observed in patient tumors (138). Although genetically engineered variants (e.g., Trp53-and Brca1/2-deficient ID8 models) improve alignment with HGSC-relevant genotypes, these systems still lack the full spectrum of human tumor heterogeneity and evolution (138). Furthermore, murine immune systems differ substantially from human immunity in cytokine signaling, immune cell composition, and checkpoint biology, which can influence therapeutic responses and limit direct translational extrapolation (145, 146).

Thus, syngeneic models should be viewed primarily as platforms optimized for interrogating immunologic mechanisms and therapeutic responses in an immune-competent setting, rather than as high-fidelity models of human tumor biology. Optimal use of these models involves pairing them with complementary human-based systems, such as patient-derived organoids or PDX models, to balance immunologic insight with molecular and clinical relevance.

### Genetically engineered mouse models (GEMMs) of ovarian cancer

Although more challenging, time-consuming, and expensive to develop, genetically engineered mouse models (GEMMs) offer several advantages over cell lines, organoids, PDX, and syngeneic models, including natural tumorigenesis, selective amplification of specific genetic aberrations, immune selection, and tumor evolution in response to chemotherapy. Transgenic mice provide a platform for researchers to investigate how genetic alterations in any tissue or cell type affect the entire organism, a context that is missing in both cell lines and organoids. They are also more effective in accurately mimicking tumor heterogeneity and the complex interactions between tumor cells and the tumor microenvironment. Most importantly, GEMMs have allowed us to understand how specific factors impact the initiation and natural progression of cancer. Thus, in addition to being useful for preclinical studies, transgenic mouse models have been instrumental in understanding the pathobiology of ovarian cancer and how the tumor microenvironment and systemic factors influence disease progression.

Several GEMMs of ovarian cancer have been developed, with different genetic alterations engineered to drive tumorigenesis and the cells in which these alterations are targeted. While early models focused on inducing genetic changes in the ovarian surface epithelial cells, an improved understanding of the cell of origin for HGSC necessitated a shift towards developing models in which the genetic alterations are directed to the fallopian tube epithelium. Consistent with genomic and protein expression data from patients with HGSC, these models involve disruption of key tumor suppressor genes, such as *p53*, *PTEN*, and *BRCA1*, and overexpression of oncogenes, such as *MYC* and *PI3KCA*. Disruption of these genes in different combinations has provided a deeper understanding of how they affect both early- and late-stage disease progression. Many of these models employ a Cre-recombinase-dependent approach to target these genomic alterations to either the ovarian surface or fallopian tube epithelium. While some models relied on intra-bursal injection of viral particles to express Cre-recombinase in the ovarian surface epithelium, others used ovarian surface epithelium and fallopian tube epithelium-specific promoters to drive Cre expression. Although the intra-bursal injection strategy is effective and comparatively easy to perform, it is limited in specificity. For instance, Cre expression in fallopian tube epithelium and ovarian stromal cells is likely to occur beyond its intended expression in the ovarian surface epithelium. Furthermore, the effectiveness of this strategy depends on the quality of the viral preparation and the technique used by the personnel performing the intra-bursal injection. These limitations can be overcome by using cell-type-specific promoters to drive Cre expression.

Common cell-type-specific promoters used to drive Cre expression in ovarian cancer models include Amhr2 (anti-Müllerian hormone receptor type 2), PAX8 (Paired box 8), and Ovgp1 (oviductal glycoprotein 1). Amhr2 is expressed primarily in the mouse ovarian surface epithelium, but low levels of Amhr2 promoter activity are observed in the fallopian tubes, skeletal muscles, and lungs. In addition to the secretory fallopian tube epithelium, PAX8 is also expressed in the kidney, thyroid gland, and uterine epithelial cells in female mice (4, 147). Due to its specificity for oviductal secretory epithelial cells, the OVGP1 promoter has become an attractive option for directing Cre expression in HGSC GEMMs (148). The strain of mice used also impacts the phenotypes observed in GEMMs. For instance, Szabova et al. (149) report no tumors upon p53 deletion in FVB mice, whereas Clark-Knowles et al. (150) report that p53 loss in FVB/n; 129SV mixed background results in the formation of leiomyosarcomas.

As GEMM models retain an intact immune system, they are also valuable for studying the tumor immune microenvironment and the effectiveness of immune-targeting strategies. The different GEMM models vary in their oncogenic drivers and promoters and, therefore, exhibit differences in the expression of antigens and proteins that modulate immune responses (151). These differences should be considered when selecting models for experiments and when interpreting data. Despite their utility in studying immune responses, GEMM models have certain weaknesses that have limited their use as a tool for testing immunotherapeutic efficacy. A major challenge with these models is that tumors develop in them over a period, and there is limited control over the time of tumor onset as well as its size. This poses a logistical challenge for designing therapeutic studies. Furthermore, because many of these GEMMs are generated on mixed backgrounds, generating transplantable syngeneic lines has also been challenging. This can be addressed by backcrossing these models, which has been done in some cases.

Several excellent reviews on GEMMs in ovarian cancer discuss the strengths and limitations of the different models (152–156). Goals of specific experiments should guide the choice of the appropriate GEMM for studies. To help investigators assess the suitability of different models for their experiments, key features of several GEMMs for ovarian cancer are presented in Table 5.

**TABLE 5:** Summary of key features of different GEMMs of ovarian cancer. *Note: although the list focuses on mouse models of high-grade serous carcinomas, some models that resulted in non-high-grade serous carcinoma tumors are included to highlight the impact of differences in mouse genetic background on phenotypes. We acknowledge that this list is not exhaustive.*

Genetic alteration	How is Cre expressed?	Target cells	Mouse strain	Tumor type	Reference
p53^−/−^ Rb^−/-^	Intra-bursal injection of retroviral Cre	Potentially ovarian surface and fallopian tube epithelium	FVB/n	Metastatic high-grade serous carcinomas	Flesken-Nikitin et al., cancer res. 2003 (166).
SV40 T-antigen (Driven by Amhr2 promoter)	Not applicable	Potentially ovarian surface and fallopian tube epithelium	C57BL/6	Poorly differentiated carcinomas	Connolly DC et al., Ca cer res. 2003 (167).
Pten^−/−^ Kras^G12D^	Intra-bursal injection of adenoviral Cre	Potentially ovarian surface and fallopian tube epithelium	C57BL6/129Sv Mixed background	Pre-neoplastic lesions; endometrioid	Dinulescu DM et al., nature med. 2005 (168).
p53^−/−^ Rb^−/-^ Brca1/2^−/−^	Intra-bursal injection of adenoviral Cre	Potentially ovarian surface and fallopian tube epithelium	FVB	Metastatic high-grade serous carcinomas	Szabova et al., cancer res. 2012 (149).
p53^R172H/+^ Rb^−/-^ BRCA1/2^−/−^					
Dicer^−/−^ Pten^−/−^	Amhr2 promoter	Stromal cells in the fallopian tube	C57BL6/129Sv Mixed background	Metastatic high-grade serous carcinomas	Kim J et al., PNAS. 2012 (169).
Brca1^−/−^ Pten^−/−^ p53^−/−^	PAX8 promoter	Fallopian tube epithelium	C57BL/6 mixed background	Metastatic high-grade serous carcinomas	Perets R et. Al., cancer cell. 2013 (4).
SV40 large T-antigen	Ovgp1-promoter	Fallopian tube epithelium	C57BL/6	Serous tubal intraepithelial carcinoma-like lesions in the oviduct and invasive adenocarcinoma in ovary	Sherman-Baust CA et al., J Pathol. 2014 (170).
Brca1^−/−^ p53^−/−^ Rb^−/-^	Ovgp1-promoter (iCreER^T2^)	Fallopian tube epithelium	B6.FVB Mixed background	Serous tubal intraepithelial carcinoma-like lesions in the oviduct and high-grade serous carcinoma (There are differences in timing of tumor development and progression and morphology of the lesions between the different genotypes)	Zhai Y et. Al., J Pathol. 2017 (171).
Brca1^−/−^ p53^−/−^ Nf1^−/−^					
Brca1^−/−^ p53^−/−^ Rb^−/-^ Nf1^−/−^					
T121 (N-term SV40 T-antigen) p53^R172H/+^ (T121 inactivates Rb)	PAX8-rtTA	Fallopian tube epithelium	B6.FVB Mixed background	High-grade serous carcinomas (Fallopian tube epithelium- and ovarian surface epithelium-derived tumors differ in latency, metastatic behavior, and other molecular features)	Zhang S et al., Nat Commun. 2019 (172).
T121 (N-term SV40 T-antigen) p53^R172H/+^ (T121 inactivates Rb)	Lgr5-Cre^ERT2^	Ovarian surface epithelium			
Dicer^−/−^ Pten^−/−^ p53^R172H/+^	Amhr2 promoter	Stromal cells in the fallopian tube	C57BL6/129Sv Mixed background	Metastatic high-grade serous carcinomas	Kim O et al., PLoS genet. 2020 (173).
p53^R273H^ MYC (Driven by Ovgp1 promoter)	Not applicable	Fallopian tube epithelium	C57BL6	Metastatic high-grade serous carcinomas (also developed uterine serous carcinoma)	Blackman A et al., cancer res Commun. 2024 (174).

### Overall limitations of *in vivo* models

While specific limitations are referenced above, *in vivo* modeling is resource-intensive, so understanding the broad limitations of each model is critical to ensure the selection of the correct model for the scientific question. PDX models are derived from patient tumors and thus can be difficult to engraft and require immune-suppressed mice, limiting evaluation of the tumor microenvironment. Humanized PDX models have evolved to better recapitulate the stroma and immune cell infiltrates but remain limited by the allogeneic nature of the immune compartment. In contrast, syngeneic models allow experimentation in the setting of an intact immune system but share similar limitations related to cell line selection, including genetic drift and molecular homogeneity. Finally, GEMMs are primarily limited by the resources and skills required for development.

### Consideration of non-genetic tumor-context features

Features such as hypoxia, oxidative stress, metabolism, and inflammatory signaling are critical determinants of HGSC progression and therapeutic response (16, 157, 158) but are variably captured across model systems. Hypoxia and nutrient gradients, which drive HIF1α signaling, metabolic rewiring, and therapy resistance, are best modeled in tumor slices and organ-on-chip systems that preserve spatial architecture or enable controlled oxygen and nutrient gradients. Oxidative stress and redox signaling, which influence DNA damage responses and platinum sensitivity, can be interrogated in both organoids and microfluidic systems, particularly when coupled with physiologic media or dynamic perfusion. In contrast, adipocyte-driven lipid metabolism, central to omental metastasis, is most effectively modeled using omental co-culture systems, where tumor cells engage in fatty acid uptake and β-oxidation to support rapid growth (127, 159).

Inflammatory cytokine networks and anoikis resistance, hallmarks of ascites-associated dissemination, are best captured by ascites-derived spheroids and organoid systems that retain multicellular aggregates and cytokine-rich microenvironments. These models enable interrogation of IL-6, TNFα, and other inflammatory mediators that promote tumor survival and immune evasion. Additionally, coagulation and protease signaling linked to thrombin activation, fibrin deposition, and metastatic niche formation require more complex systems incorporating stromal and vascular components, such as organ-on-chip platforms or *in vivo* models. Therapy-induced inflammatory remodeling, including macrophage polarization and cytokine rewiring following chemotherapy, is most effectively studied in immune-competent settings such as syngeneic models or, with important caveats, humanized PDX systems.

Collectively, these considerations highlight that no single model can recapitulate the full spectrum of non-genetic tumor-context biology in HGSC. Instead, model selection should be guided by the specific biological process under investigation: tumor slices and organ-on-chip systems for hypoxia and spatial gradients; omental co-cultures for adipocyte–tumor metabolic coupling; ascites-derived spheroids for inflammatory and anoikis-resistant states; and syngeneic or humanized models for immune, coagulation, and therapy-induced remodeling. Integrating these complementary platforms will be essential to capturing the dynamic, context-dependent biology underlying HGSC progression and therapeutic response.

## Conclusions

HGSC model selection must be driven by the biological question rather than by convenience or historical precedent. The selection of ovarian cancer models used in research is often a focus of reviewer criticism, and this review summarizes recent developments in HGSC models and provides practical guidelines to optimize model selection for a given scientific question (Table 6). Tumor initiation is best addressed using normal fallopian tube epithelium/ovarian surface epithelium cultures, CRISPR-engineered precursor organoids, and GEMMs, as these systems allow defined genetic perturbations to be introduced into candidate cells of origin and to be followed during transformation. Studies focused on therapeutic response, particularly immunotherapy, and the tumor microenvironment are best performed in models with an intact or reconstructed immune compartment. Syngeneic murine models provide the most practical platform for testing immune mechanisms in an immune-competent host, whereas humanized PDX and humanized immune-stroma PDX models offer more translational, albeit technically complex, systems for studying human tumor–immune interactions. Drug screening is best suited to scalable, patient-relevant platforms, including molecularly validated HGSC cell lines for high-throughput mechanistic studies and patient-derived organoids for patient-specific drug sensitivity testing. Therapy resistance studies require complementary approaches, including matched sensitive/resistant cell-line derivatives, preferably generated *in vivo*, and CRISPR screens for mechanistic discovery, PDOs for adaptive drug-response phenotypes, tumor slices for short-term spatial pharmacodynamic readouts, and PDX, GEMM, or syngeneic models when resistance involves stromal, immune, pharmacokinetic, or evolutionary pressures. Thus, no single model fully captures the molecular, histologic, immune, and microenvironmental complexity of HGSC; the most rigorous studies will integrate orthogonal platforms that balance molecular fidelity, physiologic relevance, scalability, and translational applicability.

**TABLE 6:** HGSC Models Matrix. PDX = patient-derived xenograft, GEMM = genetically engineered mouse models, GVHD = Graft versus host disease, HLA = human leukocyte antigen, TME = tumor microenvironment. Repro. = Reproducibility.

Model system	Molecular fidelity to HGSC	Immune Competence	Repro.	Scalability	Cost	Technical complexity	Translational relevance	Strengths	Limitations
Cell Lines (2D/3D)	Moderate–High (select lines)	None	High	High	Low	Low	Moderate	Genetically tractable; high-throughput screening	Lack TME; drift; limited heterogeneity
Patient-Derived Organoids (PDOs)	High (retain genomics)	Limited (can be co-cultured)	Moderate	Moderate	Moderate	Moderate –High	High	Capture heterogeneity; predictive drug response	Limited immune/ vascular components; selection bias
Ascites Spheroids	Moderate–High	Limited	Moderate	Moderate	Low– Moderate	Moderate	High (metastasis biology)	Model anoikis resistance, inflammation	Limited structure; short-term culture
Tumor Slices	High	Moderate (short-term preserved)	Moderate	Low	Moderate	Moderate	High	Preserve spatial architecture and TME	Short viability; no immune recruitment
Organ-on-Chip	Moderate–High	Configurable	Low– Moderate	Low	High	High	High	Models gradients, vasculature, flow	Complex; limited standardization
PDX Models	High (genomic fidelity)	None	Moderate	Low	High	High	High	Patient-specific tumor biology	Murine stroma; no immune system
Humanized PDX	High (tumor) + partial immune	Partial (incomplete)	Low– Moderate	Low	Very High	Very High	High	Human immune-tumor interactions	GVHD; HLA mismatch; cytokine incompatibility
Syngeneic Models	Low–Moderate	High	High	High	Low– Moderate	Moderate	Moderate	Fully intact immune system	Limited HGSC fidelity
GEMMs	High (engineered)	High	Moderate	Low	High	Very High	High	Model initiation/progression	Long latency; resource intensive
