# Supplementary material for: Models of high-grade serous carcinoma of tubo-ovarian origin
Source: Oncol Rev. 2026 Jul 15;20:1849834. doi: 10.3389/or.2026.1849834 (PMC13416685; doi:10.3389/or.2026.1849834)
Supplement: Supplementary file 2 [file Table1.DOCX]

**Supplemental Table 1**. Ovarian cancer cell lines and their designations. RRID = Research Resource Identifiers. TP53 status was collected from Cellosaurus and DepMap. HGSC Line Classification – Y = Recommended; C = Conditional; N = Not Recommended.

| **Supplemental Table 1** |  |  |  |  |  |  |  |
| --- | --- | --- | --- | --- | --- | --- | --- |
| **Cell Line** | **Publication Defined Histotype** | **Ref.** | **Year Published** | **RRID** | **TP53 Status** | **Cellosaurus**  **Notes** | **HGSC Line Classification** |
| OV2008 | Aytpical Non-Serous (Clear Cell /Endometrioid) | Anglesio | 2013 | CVCL_0473 | Wildtype | Contaminated. Shown to be a ME-180 derivative (PubMed=22710073). Originally thought to originate from a patient with an ovarian serous carcinoma. | N |
| A2774 | Endometrioid | Beaufort | 2014 | CVCL_U376 | Wildtype |  | N |
| 59M | Dedifferentiated Endometrial Carcinoma | Adams | 2025 | CVCL_2291 | p.His193Lysfs*49 |  | C |
|  | LGSC | Barnes | 2021 |  |  |  |  |
|  | Mixed Histology | Beaufort | 2014 |  |  |  |  |
|  | Possibly HGSC | Domcke | 2013 |  |  |  |  |
| A2780 | Endometrioid | Anglesio | 2013 | CVCL_0134 | Wildtype |  | N |
|  | Endometrioid | Barnes | 2021 |  |  |  |  |
|  | Clear Cell/Endometrioid | Beaufort | 2014 |  |  |  |  |
|  | Unlikely HGSC | Domcke | 2013 |  |  |  |  |
|  | Clear Cell/Endometrioid | Beaufort | 2014 |  |  |  |  |
| A2780 ADR (ECACC) | Clear Cell/Endometrioid | Beaufort | 2014 | CVCL_1941 | Wildtype |  | N |
| A2780 CIS (ECACC) | Clear Cell/Endometrioid | Beaufort | 2014 | CVCL_1942 | Wildtype |  | N |
| BIN67 | SCCOHT | Adams | 2025 | CVCL_S987 | Wildtype |  | N |
| CAOV3 | HGSC | Adams | 2025 | CVCL_0201 | p.Gln136Ter (c.406C>T) |  | Y |
|  | HGSC | Anglesio | 2013 |  |  |  |  |
|  | HGSC | Barnes | 2021 |  |  |  |  |
|  | HGSC | Beaufort | 2014 |  |  |  |  |
|  | Likely HGSC | Domcke | 2013 |  |  |  |  |
| CAOV4 | HGSC | Adams | 2025 | CVCL_0202 | p.Val147Asp (c.440T>A) |  | Y |
|  | HGSC | Barnes | 2021 |  |  |  |  |
|  | HGSC | Beaufort | 2014 |  |  |  |  |
|  | Likely HGSC | Domcke | 2013 |  |  |  |  |
| COLO704 | HGSC | Anglesio | 2013 | CVCL_1994 | p.Lys382Asnfs*40 (c.1146delA) |  | Y |
|  | Unlikely HGSC | Domcke | 2013 |  |  |  |  |
| COLO720E | HGSC | Anglesio | 2013 | CVCL_1995 | p.Ala138Val (c.413C>T);p.Lys373Argfs*49 (c.1118delA) | Partially contaminated. Some stocks are contaminated by COLO 704 | C |
| COV318 | HGSC | Barnes | 2021 | CVCL_2419 | p.Ile195Phe (c.583A>T) |  | Y |
|  | HGSC | Beaufort | 2014 |  |  |  |  |
|  | Likely HGSC | Domcke | 2013 |  |  |  |  |
| COV362 | HGSC | Adams | 2025 | CVCL_2420 | p.Tyr220Cys (c.659A>G) |  | Y |
|  | HGSC | Barnes | 2021 |  |  |  |  |
|  | Endometrioid/HGSC? | Beaufort | 2014 |  |  |  |  |
|  | Likely HGSC | Domcke | 2013 |  |  |  |  |
| COV362.4 | Endometrioid/HGSC? | Beaufort | 2014 | CVCL_2421 | p.Tyr220Cys (c.659A>G) |  | C |
| COV413A | Clear Cell/Endometrioid | Beaufort | 2014 | CVCL_2422 | p.Arg342Ter (c.1024C>T) |  | N |
| COV413B | Clear Cell/Endometrioid | Beaufort | 2014 | CVCL_2423 | Simple; p.Arg342Ter (c.1024C>T) |  | N |
| COV434 | SCCOHT | Karnezis | 2023 | CVCL_2010 | Wildtype | Misclassified. Originally thought to be an ovarian granulosa cell tumor but seems to be a small cell carcinoma of the ovary, hypercalcemic type | N |
|  | Unlikely HGSC | Domcke | 2013 |  |  |  |  |
| COV504 | Serous | Beaufort | 2014 | CVCL_2424 | p.Leu323Glyfs*13 (c.966_967delAC) |  | Y |
|  | Possibly HGSC | Domcke | 2013 |  |  |  |  |
| COV644 | Mucinous | Barnes | 2021 | CVCL_2425 | Wildtype |  | N |
|  | Mucinous | Beaufort | 2014 |  |  |  |  |
|  | Possibly HGSC | Domcke | 2013 |  |  |  |  |
| EFO21 | Clear Cell | Adams | 2025 | CVCL_0029 | p.Cys124Arg (c.370T>C) |  | C |
|  | Clear Cell | Barnes | 2021 |  |  |  |  |
|  | Possibly HGSC | Domcke | 2013 |  |  |  |  |
| EFO27 | Endometrioid | Adams | 2025 | CVCL_1192 | p.Arg273Cys (c.817C>T) |  | N |
|  | Endometrioid | Barnes | 2021 |  |  |  |  |
|  | Unlikely HGSC | Domcke | 2013 |  |  |  |  |
| ES2 | Dedifferentiated Endometrial Carcinoma | Adams | 2025 | CVCL_3509 | p.Ser241Phe (c.722C>T) | Probably misclassified. | C |
|  | Aytpical Non-Serous (Clear Cell/Endometrioid) | Anglesio | 2013 |  |  |  |  |
|  | LGSC | Barnes | 2021 |  |  |  |  |
|  | Clear Cell | Beaufort | 2014 |  |  |  |  |
|  | Possibly HGSC | Domcke | 2013 |  |  |  |  |
| FUOV1 | HGSC | Barnes | 2021 | CVCL_2047 | p.His179Asp (c.535C>G) |  | Y |
|  | Possibly HGSC | Domcke | 2013 |  |  |  |  |
| HEYA8 | HGSC | Anglesio | 2013 | CVCL_8878 | Wildtype |  | C |
|  | LGSC | Barnes | 2021 |  |  |  |  |
|  | Unlikely HGSC | Domcke | 2013 |  |  |  |  |
| HOC7 | LGSC/HGSC? | Beaufort | 2014 | CVCL_5455 | p.Cys275Phe (c.824G>T) |  | C |
| HS571T | Possibly HGSC | Domcke | 2013 | CVCL_0802 | p.Arg175His (c.524G>A) |  | Y |
| IGROV1 | Mixed Histology | Adams | 2025 | CVCL_1304 | p.Ser90Leufs*59 (c.267dupC) (c.267_268insC) p.Tyr126Cys (c.377A>G) |  | N |
|  | Endometrioid | Anglesio | 2013 |  |  |  |  |
|  | Clear Cell | Barnes | 2021 |  |  |  |  |
|  | Mixed Histology | Beaufort | 2014 |  |  |  |  |
|  | Unlikely HGSC | Domcke | 2013 |  |  |  |  |
| JHOC-5 | Clear Cell | Adams | 2025 | CVCL_4640 | Wildtype |  | N |
|  | Clear Cell | Anglesio | 2013 |  |  |  |  |
|  | Clear Cell | Barnes | 2021 |  |  |  |  |
|  | Possibly HGSC | Domcke | 2013 |  |  |  |  |
| JHOC-7 | Clear Cell | Anglesio | 2013 | CVCL_4641 | Wildtype |  | N |
| JHOC-9 | Clear Cell | Adams | 2025 | CVCL_4643 | Wildtype |  | N |
|  | Clear Cell | Anglesio | 2013 |  |  |  |  |
| JHOM1 | LGSC | Barnes | 2021 | CVCL_4644 | p.Arg213Ter (c.637C>T) |  | C |
|  | Possibly HGSC | Domcke | 2013 |  |  |  |  |
| JHOM2B | Mucinous | Barnes | 2021 | CVCL_4645 | p.Cys275Phe (c.824G>T) |  | C |
|  | Possibly HGSC | Domcke | 2013 |  |  |  |  |
| JHOS2 | HGSC | Adams | 2025 | CVCL_4647 | c.767_782+4delTGACCTGGAGTCTTCCAGTG |  | Y |
|  | HGSC | Barnes | 2021 |  |  |  |  |
|  | Likely HGSC | Domcke | 2013 |  |  |  |  |
| JHOS4 | HGSC | Adams | 2025 | CVCL_4649 | p.Val147Gly (c.440T>G) |  | Y |
|  | HGSC | Barnes | 2021 |  |  |  |  |
|  | Likely HGSC | Domcke | 2013 |  |  |  |  |
| Kuramochi | HGSC | Adams | 2025 | CVCL_1345 | p.Asp281Tyr (c.841G>T) |  | Y |
|  | HGSC | Anglesio | 2013 |  |  |  |  |
|  | HGSC | Barnes | 2021 |  |  |  |  |
|  | Likely HGSC | Domcke | 2013 |  |  |  |  |
| MCAS | Mucinous | Anglesio | 2013 | CVCL_3020 | Wildtype |  | N |
|  | Mucinous | Barnes | 2021 |  |  |  |  |
|  | Unlikely HGSC | Domcke | 2013 |  |  |  |  |
| NIHOVCAR3 | HGSC | Adams | 2025 | CVCL_0465 | p.Arg248Gln (c.743G>A) |  | Y |
|  | HGSC | Anglesio | 2013 |  |  |  |  |
|  | HGSC | Barnes | 2021 |  |  |  |  |
|  | HGSC | Beaufort | 2014 |  |  |  |  |
|  | Possibly HGSC | Domcke | 2013 |  |  |  |  |
| OAW28 | HGSC | Barnes | 2021 | CVCL_1614 | p.Pro152Argfs*18 (c.455delC) |  | Y |
|  | HGSC | Beaufort | 2014 |  |  |  |  |
|  | Likely HGSC | Domcke | 2013 |  |  |  |  |
| OAW42 | Clear Cell | Adams | 2025 | CVCL_1615 | Wildtype |  | N |
|  | Clear Cell | Barnes | 2021 |  |  |  |  |
|  | Serous | Beaufort | 2014 |  |  |  |  |
|  | Unlikely HGSC | Domcke | 2013 |  |  |  |  |
| OC314 | Endometrioid | Barnes | 2021 | CVCL_1616 | p.Arg273His (c.818G>A) |  | N |
| OC316 | Unlikely HGSC | Domcke | 2013 | CVCL_1618 | p.Arg273His (c.818G>A) | Contaminated. Identical to OC 314 according to STR profiling done at ICLC, to MSI data (PubMed=15677628) and to omic data (DepMap). | N |
| ONCODG1 | HGSC | Barnes | 2021 | CVCL_1882 | p.Arg248Gln (c.743G>A) | Contaminated. Shown to be a OVCAR-3 derivative (PubMed=20143388). Originally thought to originate from a 49 year old female patient with a thyroid gland papillary carcinoma. | C |
|  | Possibly HGSC | Domcke | 2013 |  |  |  |  |
| OV17R | HGSC | Beaufort | 2014 | CVCL_2672 | p.Val216Met (c.646G>A) |  | Y |
| OV56 | LGSC | Barnes | 2021 | CVCL_2673 | p.Lys101_Thr102fs (c.303_304delAA) |  | N |
|  | Clear Cell/Endometrioid | Beaufort | 2014 |  |  |  |  |
|  | Unlikely HGSC | Domcke | 2013 |  |  |  |  |
| OV7 | LGSC | Barnes | 2021 | CVCL_2675 | p.Ile254Ser (c.761T>G) |  | N |
|  | Mixed Histology | Beaufort | 2014 |  |  |  |  |
| OV90 | Mucinous | Adams | 2025 | CVCL_3768 | Simple; p.Ser215Arg (c.643A>C) |  | C |
|  | Unclassified | Anglesio | 2013 |  |  |  |  |
|  | Mucinous | Barnes | 2021 |  |  |  |  |
|  | HGSC | Beaufort | 2014 |  |  |  |  |
|  | Possibly HGSC | Domcke | 2013 |  |  |  |  |
| OVCAR4 | HGSC | Adams | 2025 | CVCL_1627 | p.Leu130Val (c.388C>G) |  | Y |
|  | HGSC | Anglesio | 2013 |  |  |  |  |
|  | HGSC | Barnes | 2021 |  |  |  |  |
|  | Likely HGSC | Domcke | 2013 |  |  |  |  |
| OVCAR5 | HGSC | Anglesio | 2013 | CVCL_1628 | Wildtype | Possibly misclassified. | C |
| OVCAR8 | Dedifferentiated Endometrial Carcinoma | Adams | 2025 | CVCL_1629 | Simple; c.376-1G>A (p.Tyr126_Lys132del, c.376_396del21) |  | C |
|  | HGSC | Anglesio | 2013 |  |  |  |  |
|  | LGSC | Barnes | 2021 |  |  |  |  |
|  | Possibly HGSC | Domcke | 2013 |  |  |  |  |
| OVISE | Mixed Histology | Adams | 2025 | CVCL_3116 | Wildtype |  | N |
|  | Aytpical Non-Serous (Clear Cell /Endometrioid) | Anglesio | 2013 |  |  |  |  |
|  | Clear Cell | Barnes | 2021 |  |  |  |  |
|  | Unlikely HGSC | Domcke | 2013 |  |  |  |  |
| OVK18 | Dedifferentiated Endometrial Carcinoma | Adams | 2025 | CVCL_3770 | p.Pro152Argfs*18 (c.455delC) |  | N |
|  | Endometrioid | Barnes | 2021 |  |  |  |  |
|  | Unlikely HGSC | Domcke | 2013 |  |  |  |  |
| OVKATE | HGSC | Barnes | 2021 | CVCL_3110 | p.Arg282Trp (c.844C>T) |  | Y |
|  | Likely HGSC | Domcke | 2013 |  |  |  |  |
| OVMANA | Clear Cell | Adams | 2025 | CVCL_3111 | Wildtype |  | N |
|  | Clear Cell | Anglesio | 2013 |  |  |  |  |
|  | Clear Cell | Barnes | 2021 |  |  |  |  |
|  | Unlikely HGSC | Domcke | 2013 |  |  |  |  |
| OVSAHO | HGSC | Adams | 2025 | CVCL_3114 | p.Arg342Ter (c.1024C>T) |  |  |
|  | HGSC | Barnes | 2021 |  |  |  | Y |
|  | Likely HGSC | Domcke | 2013 |  |  |  |  |
| OVSAYO | HGSC | Anglesio | 2013 | CVCL_3115 | p.Arg249Met (c.746G>T) | Misclassified. Originally thought to be an ovarian clear cell adenocarcinoma but shown to be a HGSC (PubMed= 24023729) | Y |
| OVTOKO | Clear Cell | Adams | 2025 | CVCL_3117 | Wildtype |  |  |
|  | Clear Cell | Anglesio | 2013 |  |  |  | N |
|  | Clear Cell | Barnes | 2021 |  |  |  |  |
|  | Unlikely HGSC | Domcke | 2013 |  |  |  |  |
| PEA1 | HGSC | Adams | 2025 | CVCL_2682 | p.Cys242Alafs*5 (c.723delC) (p.S241fs) |  | Y |
|  | HGSC | Beaufort | 2014 |  |  |  |  |
| PEA2 | HGSC | Adams | 2025 | CVCL_2683 | p.Cys242Alafs*5 (c.723delC) (p.S241fs) | Orignate from same individual as PEA1 | Y |
|  | HGSC | Beaufort | 2014 |  |  |  |  |
| PEO1 | HGSC | Adams | 2025 | CVCL_2686 | p.Gly244Asp (c.731G>A) |  | Y |
|  | HGSC | Beaufort | 2014 |  |  |  |  |
| PEO14 | HGSC | Beaufort | 2014 | CVCL_2687 | p.Cys277Phe (c.830G>T) |  | Y |
| PEO16 | HGSC/LGSC? | Beaufort | 2014 | CVCL_2688 | N/A |  | C |
| PEO23 | HGSC | Beaufort | 2014 | CVCL_2689 | p.Cys277Phe (c.830G>T) | Orignate from same individual as PEO14 | Y |
| PEO4 | HGSC | Adams | 2025 | CVCL_2690 | p.Gly244Asp (c.731G>A) | Orignate from same individual as PEO1 | Y |
|  | HGSC | Beaufort | 2014 |  |  |  |  |
| PEO6 | HGSC | Adams | 2025 | CVCL_2691 | p.Gly244Asp (c.731G>A) | Orignate from same individual as PEO1 | Y |
| RMG-1 | Clear Cell | Adams | 2025 | CVCL_1662 | Wildtype |  | N |
|  | Unclassified | Anglesio | 2013 |  |  |  |  |
|  | Clear Cell | Barnes | 2021 |  |  |  |  |
|  | Unlikely HGSC | Domcke | 2013 |  |  |  |  |
| RMG-2 | Clear Cell | Anglesio | 2013 | CVCL_2803 | Wildtype |  | N |
| RMUGS | Mucinous | Barnes | 2021 | CVCL_3158 | p.Ala347Val (c.1040C>T) |  | C |
|  | Possibly HGSC | Domcke | 2013 |  |  |  |  |
| SKOV3 | Mixed Histology | Adams | 2025 | CVCL_0532 | p.Ser90Profs*33 (c.267delC) |  |  |
|  | Aytpical Non-Serous (Clear Cell /Endometrioid) | Anglesio | 2013 |  |  |  |  |
|  | Clear Cell | Barnes | 2021 |  |  |  | C |
|  | Clear Cell /Endometrioid | Beaufort | 2014 |  |  |  |  |
|  | Unlikely HGSC | Domcke | 2013 |  |  |  |  |
|  | Clear Cell /Endometrioid | Beaufort | 2014 |  |  |  |  |
| SKOV6 | HGSC | Beaufort | 2014 | CVCL_A457 | p.Arg273Cys (c.817C>T) | Contaminated. Shown to be a C-33 A derivative | C |
| SNU119 | HGSC | Barnes | 2021 | CVCL_5014 | p.Pro151Ala (c.451C>G) |  | Y |
|  | Likely HGSC | Domcke | 2013 |  |  |  |  |
| SNU8 | HGSC | Barnes | 2021 | CVCL_5096 | p.Pro142fs (c.425_426delCT) |  | Y |
|  | Possibly HGSC | Domcke | 2013 |  |  |  |  |
| SNU840 | Unlikely HGSC | Domcke | 2013 | CVCL_5100 | Wildtype |  | N |
| TOV112D | Dedifferentiated Endometrial Carcinoma | Karnezis | 2023 | CVCL_3612 | p.Arg175His (c.524G>A) |  | N |
|  | Endometrioid | Anglesio | 2013 |  |  |  |  |
|  | Endometrioid | Barnes | 2021 |  |  |  |  |
|  | Endometrioid | Beaufort | 2014 |  |  |  |  |
| TOV21G | Clear Cell | Adams | 2025 | CVCL_3613 | Wildtype |  |  |
|  | Clear Cell | Anglesio | 2013 |  |  |  |  |
|  | Clear Cell | Barnes | 2021 |  |  |  | N |
|  | Clear Cell | Beaufort | 2014 |  |  |  |  |
|  | Unlikely HGSC | Domcke | 2013 |  |  |  |  |
| TYK-nu | Dedifferentiated Endometrial Carcinoma | Adams | 2025 | CVCL_1776 | p.Arg175His (c.524G>A) |  | C |
|  | LGSC | Barnes | 2021 |  |  |  |  |
|  | Likely HGSC | Domcke | 2013 |  |  |  |  |
| TYK-nu.CPR | Dedifferentiated Endometrial Carcinoma | Adams | 2025 | CVCL_3221 | p.Arg175His (c.524G>A) | Orignate from TYK-nu | C |
| UWB1.289 | HGSC | Beaufort | 2014 | CVCL_B079 | p.R209KfsTer6 |  | Y |
| UWB1.289+BRCA1 | HGSC | Beaufort | 2014 | CVCL_B078 | p.R209KfsTer6 | Orignate from UWB1.289 | Y |
| VOA1056_CL | LGSC | Anglesio | 2013 | CVCL_V536 | Wildtype |  | N |
| VOA1072 | HGSC | Anglesio | 2013 | CVCL_V537 | p.Arg248Gln (c.743G>A) |  | Y |
| VOA1312_CL | LGSC | Anglesio | 2013 | CVCL_V538 | Wildtype |  | N |
| VOA1400_CL | HGSC | Anglesio | 2013 | CVCL_V539 | p.Glu198Ter (c.592G>T) |  | Y |
| VOA1416_CL | HGSC | Anglesio | 2013 | CVCL_V540 | Wildtype |  | C |
